# In Vitro Viability Tests of New Ecofriendly Nanosystems Incorporating Essential Oils for Long-Lasting Conservation of Stone Artworks

**DOI:** 10.3390/gels10020132

**Published:** 2024-02-06

**Authors:** Flavia Bartoli, Leonora Corradi, Zohreh Hosseini, Antonella Privitera, Martina Zuena, Alma Kumbaric, Valerio Graziani, Luca Tortora, Armida Sodo, Giulia Caneva

**Affiliations:** 1 Institute of Heritage Science, National Research Council, ISPC-CNR, 00010 Rome, Italy; flavia.bartoli@cnr.it; 2 Department of Science, University of Roma Tre, 00146 Rome, Italy; seyedhzohreh.hosseini@uniroma3.it (Z.H.); antonella.privitera@uniroma3.it (A.P.); martina.zuena@uniroma3.it (M.Z.); alma.kumbaric@uniroma3.it (A.K.); armida.sodo@uniroma3.it (A.S.); giulia.caneva@uniroma3.it (G.C.); 3 Department of Chemistry “Giacomo Ciamician”, Alma Mater Studiorum, Ravenna Campus, Bologna University, 48121 Ravenna, Italy; 4 National Institute of Nuclear Physics (INFN), Roma Tre Section, 00146 Rome, Italy; valerio.graziani@roma3.infn.it (V.G.); luca.tortora@uniroma3.it (L.T.)

**Keywords:** cultural heritage, multifunctional coating, green gels, biodeterioration, biofilms, natural biocide, oregano oil, eugenol, silica gel

## Abstract

The study explores the application of natural biocides (oregano essential oil and eugenol, directly applied in solutions or encapsulated within silica nanocapsules) for safeguarding stone cultural heritage from biodeterioration, using green algae (*Chlorococcum* sp.) and cyanobacteria (*Leptolyngbya* sp.) as common pioneer biodeteriogens. Core-shell nanocontainers were built for a controlled release of microbicidal agents, a safe application of chemicals and a prolonged efficacy. The qualitative and quantitative evaluations of biocide efficiency at different doses were periodically performed in vitro, after six scheduled intervals of time (until 100 days). The release kinetics of composite biocide-embedding silica nanocapsules were characterized by the UV-Vis spectroscopy technique. Data showed both promising potential and some limitations. The comparative tests of different biocidal systems shed light on their variable efficacy against microorganisms, highlighting how encapsulation influences the release dynamics and the overall effectiveness. Both the essential oils showed a potential efficacy in protective antifouling coatings for stone artifacts. Ensuring compatibility with materials, understanding their differences in biocidal activity and their release rates becomes essential in tailoring gel, microemulsion or coating products for direct on-site application.

## 1. Introduction

Preservation of stone cultural heritage surfaces in outdoor environments is a challenging activity for conservators, due to the influence of environmental conditions and tedaphic factors, which can promote abiotic weathering factors and damaging biological growths [1,2,3,4,5]. Cyanobacteria, green algae and sometimes also meristematic fungi and lichens are generally the first biological colonizers of stone as they are pioneer organisms that only require light, little water and few nutrients, and they can survive at the alkaline pH of stone building materials [6,7,8,9,10]. They form overall phototrophic biofilms and gelatinous patinas of different colors (brown, black, green and orange) on surfaces, which can absorb and retain water to withstand long periods of drying. These patinas cause not only aesthetic alteration, but in several cases, microorganisms grow inside the pores and crevices of the substrate, forming mixed communities of algae, cyanobacteria and fungi, leading to microcracks and bio-pitting as they are able to dissolve carbonates [8,10,11,12,13,14,15,16,17,18].

In response to this challenge, various interventions have been developed to remove the organisms responsible for degradation [19,20,21,22,23,24,25,26]. Generally, biocidal treatments or physical methods have been applied [19,20,24,25,26,27,28,29], but often they are not long lasting, as the biodeteriogens reappear on the stone after a short time if the environmental conditions remain favorable [22,26,27,28,29,30,31,32]. Biocides can be classified according to several aspects, including their chemical nature, the target organisms, the mode of action and the type of formulation; they must also meet specific requirements to be applied in the treatment of works of art, such as high efficacy against biodeteriogens, absence of interference with the materials and low toxicity to human health and the environment [33,34]. The application of traditional biocides can give rise to potential risks for human health and for the environment, demanding cautious assessment of their acute and chronic toxicity [19,20,25,27,34,35,36]. In any country, regulations exist for the marketing and use of biocides against harmful organisms, to protect humans, animals, plants and materials, such as for Europe with the “Biocidal Product Regulation”, BPR (Regulation 528/2012).

To address the challenges of reducing risks, a shift was carried out towards using “green” biocides derived from natural sources [22,23,26,29,30,31,32,33,35,37,38,39,40,41,42,43,44,45,46,47,48,49], and overall essential oils (EOs), which are constituted by a mixture of different organic molecules, such as terpenes, and then aldehydes, alcohols and esters that could be present too as minor components were used, even if only a few of them have biocidal properties [46,47,48,49]. Such green selection can be improved, when it is joined with the use of nanomaterials, which can reduce and modulate the biocidal doses. Nanoparticles, such as those made by zinc, silver, copper and titanium oxides, seem to offer alternative solutions providing antimicrobial activities and when applied in coatings, they enhance the fixation on the stone surfaces, reducing the potential environmental dispersion [19,33,50,51,52,53,54,55,56,57,58,59,60]. Ranging from inorganic nanoparticles to hybrid composites and gels or microemulsions, like titanium oxide combined with silver nanoparticles, showed enhanced efficacy against microorganisms due to their large specific surface area, controlled shape, particle size, pore diameter and biocompatibility [19,33,50,51,52,53,54,55,56,57,58]. Furthermore, especially silica nanoparticles with mesoporous structure, and their application on the stone surface incorporated in the gels, microemulsions or coating showed interesting results in providing controlled release mechanisms and such compounds can be considered safe and prominent materials in the prevention of biodeterioration [56,57,58,59,60,61].

In previous studies, we evaluated, using in vitro tests, the efficacy of silica nanocontainers (nanocapsules (NC) and mesoporous nanoparticles (MNP) containing a commercial biocide (Mercaptobenzothiazole) and a sea plant antimicrobial substance (Zosterate, ZOS) on green algal species widely occurring on stone monuments, and in parallel, we also evaluated different coatings’ formulations [61,62,63]. Such studies demonstrated that both the differential nanosystems and the biocide chemical composition can produce a differential efficacy in the antimicrobic activity against green algae. More deeply, ZOS applied as free biocide did not show relevant biocidal action against the algal cells, probably due to its capacity of preventing the adhesion of the microorganisms to the substrate, instead of killing them. Furthermore, the MNP silica nanosystems presented a relative higher efficacy than the NC ones, and this seems ascribable to their structure, characterized by hexagonally packed cylindric mesopores, in which the biocide is less trapped and thus quickly released [61,62,63]. At the same time, the synthesis of MNP resulted in being more difficult and strictly correlated to the molecular structure of the selected biocide, so NC resulted in being much more easily buildable and of potential practical use.

More recently, we also evaluated their effectiveness for a long-lasting efficacy, through in situ tests, located in favorable conditions of humidity and solar radiations [64]. The experimentations gave interesting results, and then, continuing the previous research, this ongoing study focuses on testing new stone coatings formulated with promising green biocides compatible with silica nanocapsules for a controlled release. This multifaceted approach combines green biocides and nanomaterials to ensure effective conservation strategies for stone cultural heritage to improve the sustainability and efficacy of longlasting conservation treatments while minimizing environmental and human health impacts. In this work, by in vitro tests of combined natural biocide with nanotechnology in controlled laboratory conditions, we aim to evaluate the encapsulation potentiality and biocidal efficacy of two different essential oils (oregano oil and eugenol) for preventing biological colonization.

## 2. Results and Discussion

### 2.1. SEM Characterization of the Nanocapsules

**Figure 1:**
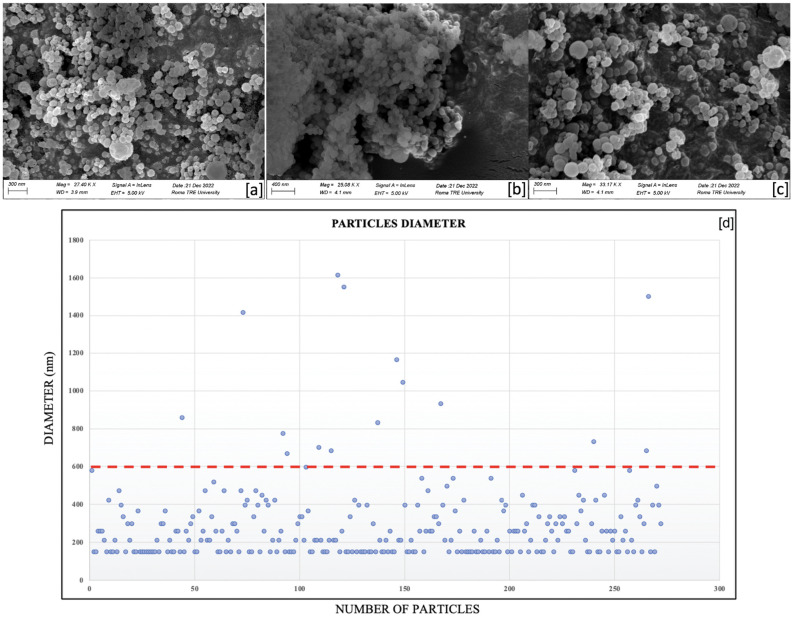
SEM images of nanocapsules, NCs: (**a**) not loaded with biocides (NC EMPTY); (**b**) loaded with oregano oil (NC OO); (**c**) loaded with eugenol (NC EU); (note the bars); (**d**) dimensional analysis of the silica nanosystems, emptied and loaded with biocides. The limit to 600 nm (red dotted line) defines the nanostructures.

Scanning electron microscopy images of secondary electrons of silica nanocapsules, empty (Figure 1a) and loaded with oregano oil (Figure 1b) and eugenol (Figure 1c), showed that the shapes and morphologies of the nanocontainers were almost identical, presenting spherical and regular shapes in each case, with no evidence of relevant differences.

Generally, the diameter distribution, elaborated through the ImageJ 1.54h software, is less than 600 nm (evidenced by a red line). Nevertheless, the densest area was represented by the portion of Figure 1d under 200 nm, meaning that the majority of nanoparticles has sizes equal to and lower than 200 nm.

The previous studies exploring natural biocides and silica nanostructured materials for preserving cultural heritage in antifouling applications revealed promising possibilities but also certain limitations [61,62,63]. Previous works on the one-step self-assembly synthesis method, involving TEOS condensation and diethyl ether outgassing, showed a consistent production of nanocontainers with comparable shapes and morphologies regardless of the integrated substances, suggesting scalability and practical application [61,65,66,67]. In the same way, by comparing the new data with results of the previous experiments on silica nanosystems loaded with the commercial biocide 2-mercaptobenzothiazole (MBT) [65], we observed that the MBT nanoparticle showed spherical and regular shapes, but with a higher average diameter with respect to the empty ones. This suggests that the dimensions of the biocide’s molecules shaped the form of the surfactant micelles, and thus, that of the silica nanocapsules, increasing their volume. In the case of the current research, instead, it could be possible that molecular weights of oregano oil constituents and eugenol did not significantly influenced the dimensions of the micelles during the synthesis process, probably due to their reduced molecular weights in comparison with the one for MBT.

### 2.2. Identification of Microorganisms—Morphological Evaluations

**Figure 2:**
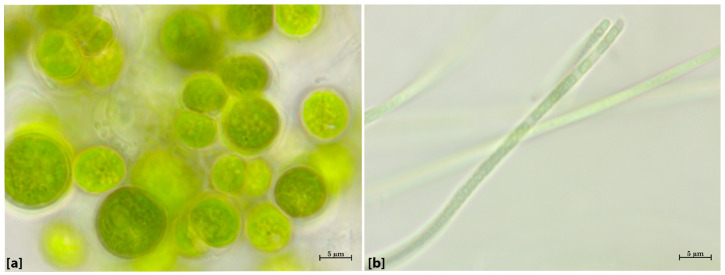
Optical microscope images of the cells (100×): (**a**) coccoidal cells of the green algae *Chlorococcum* sp.; (**b**) filamentous cells of the cyanobacteria *Leptolyngbya* sp.

Under the optical microscope (10×, 20× and 100× magnifications), two types of cellular structures have been observed: spherical/ellipsoidal cells light green/yellow colored (Figure 2a), of dimensions varying between 5 and 10 μm, and dense aggregates formed by long and thin green-colored filamentous structures (Figure 2b). The morphological analysis allowed the identification of two genera of green algae and of cyanobacteria. The spherical cells were related to the Chlorococcaceae family, and assigned to the genus *Chlorococcum* sp. The filamentous cells were related to the Leptolyngbyaceae family, and assigned to *Leptolyngbya* sp. These genera present a characteristic that is relevant for biodeterioration issues, that is their capacity to form endolithic colonization on different stone substrates.

### 2.3. UV–Vis Spectroscopy Results and Release Profiles

**Figure 3:**
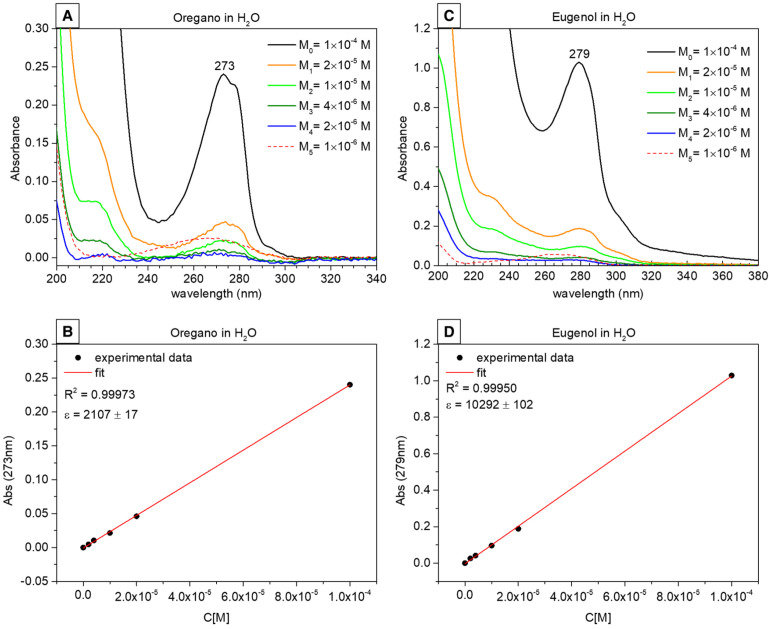
UV–Vis absorption measurements of standard solutions: (**A**) OR standard UV curves and (**B**) calibration curve obtained from the absorbance values at 273 nm; (**C**) EU standard UV curves and (**D**) calibration curves obtained from the absorbance values at 279 nm. The R^2^ factors for the data fit and the molar extinction coefficients are reported in the figures.

**Figure 4:**
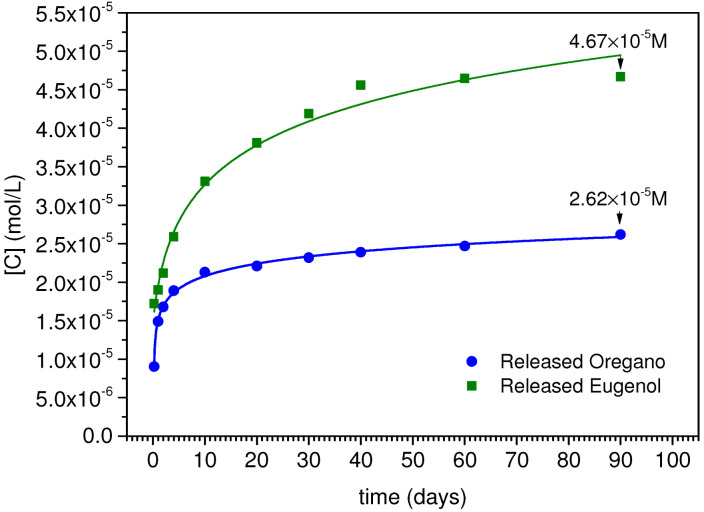
Release profiles of oregano (circle symbol) and eugenol (square symbol). The kinetic curves are represented by logarithmic fit (R^2^= 0.99657 for OO and R^2^= 0.98078 for EU).

The UV–Vis calibration curves of OO and EU standard solutions were obtained as absorbance versus concentration and we used these data to estimate the concentration of the released compounds. The UV–Vis spectrum of OO showed a main band centered at 273 nm (Figure 3A) and the relative OO Beer’s plot for a molar extinction coefficient ε_(273nm)_ = (2107 ± 17) M^−1^/cm (Figure 3B). The UV–Vis spectrum of EU showed a main band centered at 279 nm (Figure 3C) and the relative Beer’s plot showed a molar extinction coefficient ε_(279nm)_ = (10,292 ± 102) M^−1^/cm (Figure 3D). For both systems, the lowest concentration (1 × 10^−6^ M) fell below the detection instrumental limits, resulting in an artifact (dotted UV curve in Figure 3B,D). Using the protocol described in Section 4, dissolution profiles of both green biocides were obtained as concentration versus time (symbols in Figure 4) and a logarithmic fit was used to represent the kinetic curves (continuous curves in Figure 4) [68]. The OO and EU plots differed both in the quantity of released biocide and in the kinetics. In the case of oregano, an initial burst was observed, where approximately 70% of the total released biocide was measured after four days. Then, the remaining 30% of biocide was released very slowly, reaching a total amount of released oregano oil equal to 2.62 × 10^−5^ M. This release profile suggests that not all the encapsulated oregano oil has been released in the considered time interval.

In the case of eugenol, the biocide was a gradual and sustained release of up to 40 days (about 98% of the total released biocide), then a plateau was observed of up to 90 days. The total amount of released eugenol was equal to 4.67 × 10^−5^ M.

Since the same synthesis procedure has been performed, the two systems have the same nanoparticle structure and texture, thus not influencing the different release kinetics. On the other hand, the release is a function of the biocide amount encapsulated during the synthesis procedure (these aspects will be deeply described in another work in progress) and of the biocides’ water solubility. For instance, the principal compound present in oregano oil, carvacrol (60–86% of total metabolites), is moderately soluble in water (1250 mg/L at 25 °C) [69], while p-cymene and g-terpinene, the two minor compounds (up to about 10–20% of total metabolites), are nearly insoluble in water (23.4 mg/L and 8.68 mg/L at 25 °C, respectively) [70]. Eugenol is more soluble than the oregano compounds (2460 mg/L at 25 °C) [71], promoting the release and the diffusion of the biocide in vitro as well as in the environment. Regarding release data, we can conclude that the most efficient spreading system appears to be the one containing eugenol.

### 2.4. Analysis of the Efficacy of Biocides—Viability Tests

**Figure 5:**
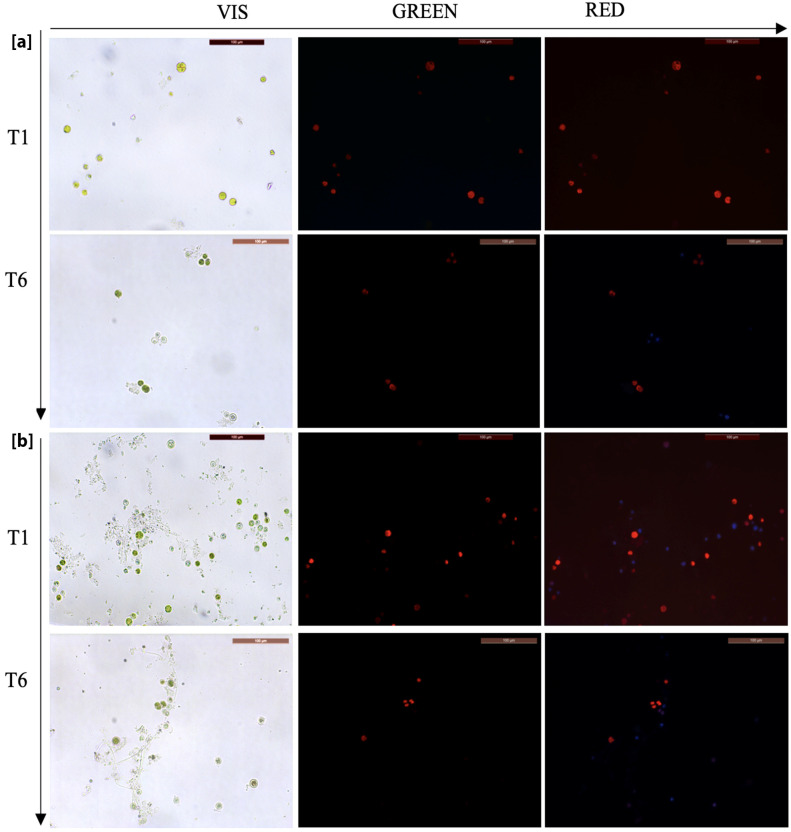
The trend of biocide efficiency observed in the six intervals of time using microscope images at 20× magnification in visible light and UV filter green and red: (**a**) NC EU 3 mg; (**b**) NC OO 3 mg (red fluorescence is an indicator of vitality).

The experiments evaluated the efficacy of silica nanocapsules, empty ones, oregano oil-loaded, eugenol-loaded, and pure biocides, oregano oil/eugenol at two different weights of nanocontainers and pure biocide (1 mg and 3 mg). It was also useful to test the silica nanocapsules empty (meaning not loaded with a biocide), because of their intrinsic biocidal property, in comparison with the silica nanocontainers loaded with green biocides. The observations of the samples under visible and fluorescence RGB filters demonstrated an antimicrobial activity of all the biocidal systems, even if displaying distinct trends in efficiency (Figure 5). In the case of the green alga *Chlorococcum,* initially, red fluorescence was prominent in cells for all types of nanocapsules. After 100 days, red cells were prevalent in tests with 1 mg, whereas those with 3 mg showed a reduced number of red cells and an increase in blue/green ones.

Pure oregano oil and eugenol were both effective, causing an evident decrease in fluorescence (red cells) under UV light, replaced by more blue/green cells. Towards the end of the experiment, few or no living cells were observable in visible or UV light, showing a total biocidal effect. Pure eugenol exhibited a decrease in fluorescent cells over time, with an increase in blue/green cells surpassing red cells. Finally, after 100 days, no living cells were observable with any filter, indicating complete efficacy. Similar trends were observed in tests with *Leptolyngbya* sp., indicating subsequent decreases in cell viability over time. In very few cases after 70–100 days from the beginning of the analysis, the filaments appeared fluorescent at the observation with the green and the red filters.

Concerning *Leptolyngbya* sp., the obtained results showed a decreasing trend of vitality very similar to that observed for *Chlorococcum* sp., especially in case of the lower quantity, whereas the application of the highest quantity showed a higher variation in reducing the biological colonization. In particular, a medium–high efficacy was observed for biocidal systems in nanocontainers, with respect to free essential oils. In the same way, the highest quantity of biocides in the silica nanocapsules seemed to be the most effective one, since red fluorescence was never observed during the entire period of analysis; the same occurred with the maximum quantity of silica nanocapsules loaded with eugenol. Indeed, in previous tests with oregano oil and eugenol as pure substances, filaments of *Leptolyngbya* sp. resulted in being quite resistant [8].

Considering the case of *Chlorococcum* sp., the cell count (Table 1) confirmed that the qualitative data obtained by the optical microscope observations and that the vitality of the cells underwent a reduction in all the biocidal systems.

However, the decline in fluorescence was not consistently linear over time, showcasing different efficacy trends among the systems. In general, as expected, the efficiency was proportional to the doses, and the silica nanocapsules with higher weight (3 mg) were able to more efficiently reduce the number of active cells due to the higher quantity of contained biocide with respect to those of lower weight (1 mg) containing lower biocide quantities. Furthermore, the nanocontainers self-showed a good antimicrobial activity, but they also gave rise to a reduction of the biocidal activity with respect to the same doses of the pure substance.

**Figure 6:**
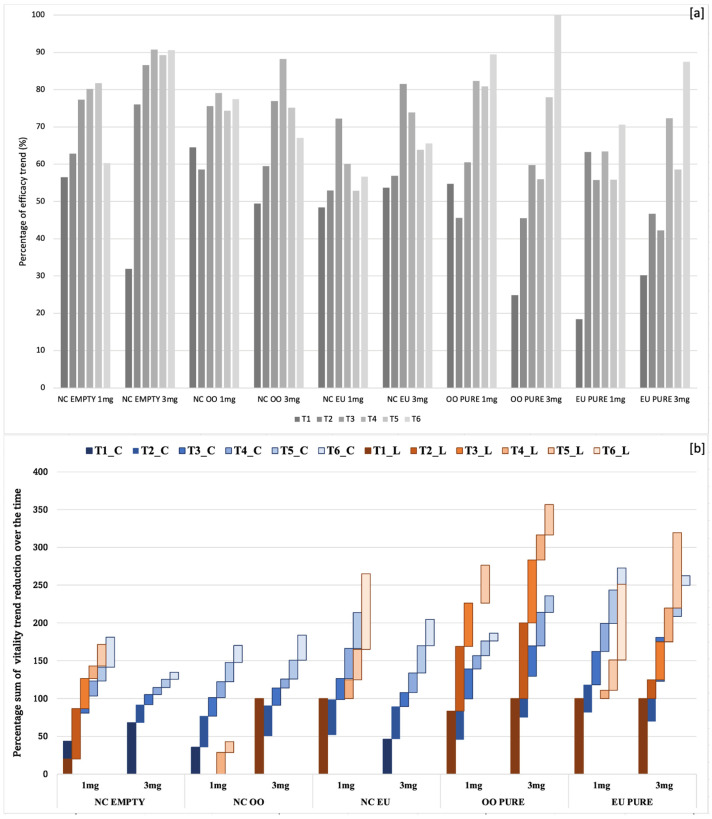
Biocidal efficiency of the different biocidal systems: (**a**) the efficacy’s trend of each biocidal system over time in the six intervals of timing (from T1 to T6 as T1 (after 10 days), T2 (after 20 days), T3 (after 30 days), T4 (after 50 days), T5 (after 70 days) and T6 (after 100 days (using only *Chlorococcum* sp. as reference organism); (**b**) the reduction of vitality trend for the two tested microorganisms, *Chlorococcum* sp. in blue and *Leptolyngbya* sp. in red (NCs = nanocapsules, OO = oregano oil; EU = eugenol).

In fact, both oregano oil and eugenol, when used as pure substances, showed more marked effects with the minimum quantity, exhibiting a more consistent and linear reduction trend over time (Figure 6a). The comparative analyses carried out between different biocidal systems, including empty nanocapsules and pure biocides, offered valuable insights into their efficacy against diverse microorganisms. These insights provide a glimpse into how encapsulation affects the release dynamics and overall effectiveness. Finally, similar decreasing paths for vitality trends were observed for the two tested taxa (Figure 6b).

Regarding *Chlorococcum* sp., it is possible to affirm that the empty silica nanocapsules (3 mg) also seemed to be an effective biocidal system, also considering its almost linear trend in the reduction of vitality within all the periods of observation, from 10 to 100 days since the beginning of the experiment. Obviously, the most effective activity was observed by using oregano oil and eugenol as free substances, while a loss of efficacy by nanocapsules seemed to occur; even these technologies guarantee a more efficient long-lasting activity. In fact, when using the same quantity, pure EOs showed the highest efficiency with respect the encapsulated nanocontainers and this proves that nanocontainers loaded with biocides need a higher quantity to work, instead, free substances can come into contact and start to exert their action against colonies almost immediately [62,72].

Specifically, oregano oil and eugenol showed a more linear decreasing trend in vitality of the cells, with respect to nanocontainers loaded with biocides that, on the contrary, presented an oscillating trend. This could be likely due to the encapsulation, which allows a gradual release of biocides from the core-shell nanostructures, producing a lower amount of biocide, with respect to the quantity of not-confined product [62]. The influence of release, which is also a function of the specific release rate, is also confirmed by the differences in efficacy trends depending on quantities of biocidal systems. In fact, the silica nanocapsules with the highest quantity produced a more marked reduction in vitality trends, with respect to the results obtained with the nanocapsules with the lowest quantity, since the loading capability of the nanocontainers influences the biocidal effectiveness of the nanosystems.

Among the nanosystems, in fact, it is noteworthy that empty silica nanocapsules (1 mg), silica nanocapsules loaded with oregano oil (3 mg) and silica nanocapsules loaded with eugenol (1 mg and 3 mg) seemed to be more effective at the very beginning of the analysis (after only 10 days), with respect to empty silica nanocapsules (3 mg), oregano oil (3 mg) and eugenol (1 mg and 3 mg). However, after 50–70 days, they seemed to lose their efficacy. This could be derived, firstly, by the release dynamics of biocides from the nanocontainers, and it was supposed that once nanocapsules have been applied, the release rate results are quite high, decreasing over time, up to the loss of their efficacy [62]. As confirmed by the release profiles, the eugenol is more soluble in water than the oregano oil [70,71], resulting in a higher rate of release and diffusion.

The research provides valuable insights into the potential of silica nanocapsules for preserving cultural heritage artifacts. These good results can be the starting point to test their application on the stone surface incorporated in the gel, microemulsion or coating [56,64,73]. However, as proven by previous studies [61,62,65], the synthesis and the nanoencapsulation step strictly depends on addressing the observed limitations, such as the fluctuating efficacy trends, which demand more extensive investigations.

## 3. Conclusions

Nanotechnology offers the possibility to reduce the quantity of biocides while enhancing the treatment effectiveness in preventing colonization. Thus, silica nano-systems can be a promising technology to preserve cultural heritage thanks to their long-lasting reliability, enabling the controlled release of biocides and an eco-friendly approach. Both of the tested essential oils (oregano oil and eugenol), such as the nanocapsules without addition of biocides, showed a good efficacy against the selected target organisms. The controlled release capabilities of silica nanocapsules indicated interesting potential applications in protective antifouling gels, microemulsions or coatings for stone artifacts. To improve the efficiency of the systems, understanding the differences in release rates of these biocides has become essential in tailoring gels, microemulsions or coating products for direct on-site application.

## 4. Materials and Methods

### 4.1. Biocidal Description

Considering some previous results from a comparative evaluation of natural compounds [22,35,40,72,74,75], we selected two natural biocides: the oregano essential oil and eugenol (C_10_H_12_O_2_: phenylpropanoid). Oregano oil, derived from *Origanum vulgare* L., a Mediterranean aromatic perennial plant of the Lamiaceae family, contains components like carvacrol, thymol, *γ*-terpinene and p-cymene; terpinene-4-ol, linalool, *β*-myrcene, trans-sabinene hydrate and *β*-caryophyllene are also present [76,77,78,79]. Recognized for its antimicrobial, antiviral and antifungal properties, oregano oil has been studied against fungi affecting stone and wooden artifacts [43,80]. Eugenol is a phenolic aromatic compound, characterized by an oily consistency and spicy aroma, extracted from different plants belonging to *Lamiaceae*, *Lauraceae*, *Myrtaceae* and *Myristicaceae* families. It can be synthesized via the allylation of guaiacol using allyl chloride or through biotransformation by various microorganisms such as *Corynebacterium* spp., *Streptomyces* spp. and *Escherichia coli* [81,82]. The tested compounds were obtained respectively by NOW foods company (https://www.nowfoods.com) (accessed on 15 January 2023) and REAGENTPLUS(R), (Eugenol 99%).

### 4.2. Nanoencapsulation Step

We synthesized silica nanocontainers, namely core-shell nanocapsules (NCs), by using an oil-in-water mini-emulsion, stabilized in the presence of cetyltrimethylammonium bromide (CTAB, Aldrich, Italy, Milan) surfactant micelles and in an alkaline environment, basically following the procedure described by Privitera et al. [83]. Some adaptations were needed during the nanoencapsulation of essential oils, particularly an increase in the stirring (1000 rpm) to avoid the coalescence of the oil drops. In two separate synthesis procedures, oregano oil and eugenol (respectively 0.01 g) were added to the oily phase (diethyl ether, 25 mL). Then, the synthesis proceeded as described in the literature, such as the characterization of the morphology, structure and texture of these loaded silica nanocontainers [83]. The silica nanocapsules were characterized through scanning electron microscopy (SEM), using a Zeiss Sigma 300 (Oberkochen, Germany) instrument, coupled with an ETSE (Everhart–Thornley secondary electron) detector. Moreover, the ImageJ software had been exploited to evaluate the diameter size distribution of the core-shell nanocapsules, by building a graphic of diameter distribution derived from the SEM images.

### 4.3. Release Study

The release study of both composite silica nanocapsules incorporating oregano essential oil (labeled as “OO”) and pure eugenol (labeled as “EU”) was performed by UV-Vis spectroscopy technique. First, calibration curves for the estimation of the chemical concentration were obtained from the UV-Vis spectra recorded on standard aqueous solutions of OO and EU at fixed concentrations (1 × 10^−4^, 2 × 10^−5^, 1 × 10^−5^, 4 × 10^−6^, 2 × 10^−6^ and 1 × 10^−6^ mol/L) [83].

Subsequently, the following procedure was used to obtain the release profiles of both nano systems in static conditions, in order to compare the release with the in vitro antimicrobial activity data: ten aliquots of each sample (nanocapsules incorporating OO and EU) were placed in Falcon tubes by dispersing 20 mg of sample powder in 5 mL of ultrapure water as dissolution medium; the aliquots were stirred at 100 rpm for 5 min to break up and homogeneously disperse the sample, after which they were left to settle. Finally, the supernatant was picked up from each aliquot at set times (6 h, 1, 2, 4, 10, 20, 30, 40, 60 and 90 days) by using a syringe equipped with a filter (Whatman, Maidstone, UK, 0.2 μm pore size) and collected in a 1 cm path length quartz cuvette for the analysis.

UV–Visible absorbance spectra were obtained by using a Thermo Scientific™ Evolution™ 350 UV–Vis Spectrophotometer(Fisher Scientific, Milan, Italy), in the wavelength range 350–190 nm, with a sampling step of 1 nm. The release profiles were obtained by evaluating the concentration of biocide dissolved in the medium versus time. All experiments were performed at room temperature.

### 4.4. Cultivation and Identification of Microorganisms

The biofilms were collected from the northern side of the Aurelian Walls in Rome, close to the Museum of the Walls, near Porta San Sebastiano. The biofilms were cultivated in the laboratory using the BG-11 solution, diluted in deionized water and exposing the cultures to natural light. Such procedure favors the growth of the photosynthetic microflora, as usually observed as a major component in such conditions, as described in previous works [45,61,62]. The morphological identification of the species was performed through microscopy observations under visible light at the magnifications of 10×, 20× and 100×, according to UNI 10923 [84], consulting the international dataset and the taxonomic identification available dichotomous keys e.g., Guiry and Guiry 2022; www.algaebase.com (accessed on 15 January 2021) [85].

### 4.5. In Vitro Tests

The evaluation of the efficacy of the biocidal systems against the selected microorganisms was conducted through viability tests, considering five different situations: silica nanocapsules not loaded with a biocide (empty NC), silica nanocapsules loaded with oregano oil (NC OO), silica nanocapsules loaded with eugenol (NC EU), oil of oregano as pure substance (OO PURE) and eugenol as pure substance (EU PURE). The nanocontainers were loaded with a concentration of biocide related to the loading capability. Empty silica nanocapsules (not loaded with a biocide) were also evaluated for their biocidal action, comparing them to the other silica nanomaterials loaded with green biocides. Indeed, they contain hexadecyltrimethylammonium bromide (CTAB), a cationic surfactant which, in bioprocesses, is active at the level of cellular membrane inhibiting the metabolic processes of the cells of microorganisms. Each system was tested using a lower (1 mg) and a higher (3 mg) weight of nanocontainers and pure substance, respectively, added to 1 mL of liquid culture (following the protocol described in [62].

**Figure 7:**
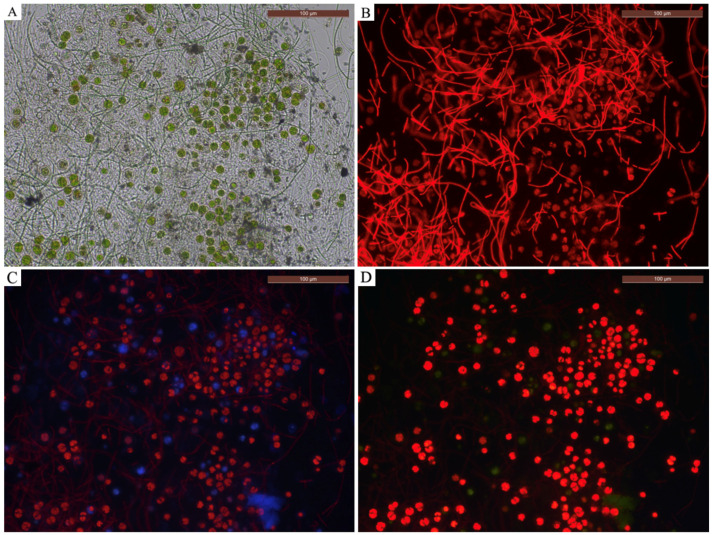
Microscope images of the algal cells captured with filters: (**A**) Vis, (**B**) green, (**C**) red, (**D**) blue.

The biocidal efficacy was periodically evaluated, after scheduled intervals of time; specifically, there were six intervals of time from one evaluation to the next one, indicated as T1 (after 10 days), T2 (after 20 days), T3 (after 30 days), T4 (after 50 days), T5 (after 70 days) and T6 (after 100 days); so, the 12 filled test tubes for each product were tested in each time control (in total 60 tubes). We observed the control and the treated cultures, in triplicate form for each product, under an optical microscope (Zeiss Axioplan 2, Rome, Italy) equipped with a photo camera (LEICA DFC 450 C, Rome, Italy) at 20× magnification in visible and auto-fluorescent light. The autofluorescence of cultured microorganisms was observed with multi-channel detection, in blue (450–500 nm), red (380–450 nm) and green (500–570 nm) wavelengths (Figure 7).

Autofluorescence was used for a qualitative–quantitative analysis, namely, as an indicator for discriminating between living cells (intense fluorescence), dying cells (slightly visible fluorescence) and dead cells (complete absence of fluorescence). Following the methodology of previous works [62], we also evaluated the loss of each cell’s fluorescence as an indicator of biocidal efficiency.

## Figures and Tables

**Table 1:** *Chlorococcum* sp. percentage of cell count responses to fluorescence for each nanosystem and RGB filter over time (T1–T6).

Time	Filter	Cells	NC EMPTY 1 mg	NC EMPTY 3 mg	NC OO 1 mg	NC OO 3 mg	NC EU 1 mg	NC EU 3 mg	OO PURE 1 mg	OO PURE 3 mg	EU PURE 1 mg	EU PURE 3 mg
**T1**	**Green**	**Red**	43.5	68.1	35.5	50.5	51.6	46.3	45.3	75.1	81.6	69.8
	**Red**	**Red**	40.8	63.3	29.9	48.5	51.1	42.6	42.1	75.9	81.6	68.5
		**Blue**	38.7	97.0	29.8	40.7	36.0	30.6	35.8	27.0	11.5	16.7
	**Blue**	**Red**	49.5	64.5	36.1	49.0	55.9	50.4	41.1	75.5	80.9	68.5
		**Green**	31.0	86.7	26.0	29.4	36.0	27.3	72.6	29.0	13.9	19.1
**T2**	**Green**	**Red**	37.2	24.0	41.4	40.5	47.1	43.1	54.4	54.5	36.8	53.3
	**Red**	**Red**	39.8	23.6	41.4	35.8	45.3	41.8	55.1	54.0	34.2	47.5
		**Blue**	40.1	51.2	35.2	43.9	36.6	41.2	42.2	41.7	41.0	36.1
	**Blue**	**Red**	37.2	27.2	44.1	43.2	51.7	44.4	46.9	50.6	34.2	47.5
		**Green**	36.6	48.0	31.7	37.2	36.0	39.2	44.9	36.2	27.4	21.3
**T3**	**Green**	**Red**	22.7	13.4	24.4	23.1	27.7	18.5	39.4	40.2	44.3	57.8
	**Red**	**Red**	22.7	14.8	26.2	24.1	27.9	17.3	48.0	57.1	41.0	52.6
		**Blue**	70.0	71.0	64.5	49.5	52.7	43.9	43.7	35.0	42.6	54.5
	**Blue**	**Red**	24.9	16.9	29.1	26.2	28.3	22.2	37.3	35.3	26.6	46.8
		**Green**	55.4	60.2	41.0	27.7	34.3	22.7	33.6	38.7	32.4	33.1
**T4**	**Green**	**Red**	19.8	9.3	20.9	11.8	39.9	26.1	17.6	44.1	36.6	27.7
	**Red**	**Red**	18.9	9.8	20.9	11.8	38.8	24.7	9.1	43.3	36.1	27.7
		**Blue**	45.4	73.2	60.1	67.0	57.7	64.0	73.9	63.5	52.3	61.5
	**Blue**	**Red**	29.8	13.4	25.8	16.8	40.3	31.8	10.8	25.1	36.6	23.1
		**Green**	30.9	55.6	33.3	47.2	39.2	37.8	44.9	56.5	25.9	36.9
**T5**	**Green**	**Red**	18.2	10.7	25.6	24.8	47.1	36.2	19.1	22.0	44.2	41.4
	**Red**	**Red**	18.2	11.4	25.0	24.1	47.1	35.7	12.8	5.1	15.5	27.6
		**Blue**	52.8	79.9	57.1	58.4	48.8	63.4	87.2	86.4	72.1	75.9
	**Blue**	**Red**	27.5	11.4	28.2	27.0	48.8	36.2	10.6	5.1	16.3	27.6
		**Green**	27.2	59.2	35.4	46.0	34.3	46.9	78.7	81.4	63.6	51.7
**T6**	**Green**	**Red**	39.7	9.4	22.5	33.0	43.4	34.5	10.5	0.0	29.4	12.5
	**Red**	**Red**	39.7	10.4	21.6	35.2	54.0	39.5	10.5	0.0	29.4	25.0
		**Blue**	55.4	89.6	73.0	48.4	28.3	55.5	89.5	90.9	52.9	62.5
	**Blue**	**Red**	42.1	22.6	24.3	39.6	66.4	42.9	10.5	0.0	26.5	12.5
		**Green**	14.9	58.5	38.7	19.8	4.4	35.3	65.8	45.5	38.2	37.5

## Data Availability

The data presented in this study are openly available in article.
